# Financial crisis, labor market frictions, and economic volatility

**DOI:** 10.1371/journal.pone.0291106

**Published:** 2023-09-28

**Authors:** Wenni Lei, Zhe Li, Dongzhou Mei

**Affiliations:** 1 School of Economics, Minzu University of China, Beijing, China; 2 School of International Trade and Economics, Central University of Finance and Economics, Beijing, China; Universiti Malaysia Sabah, MALAYSIA

## Abstract

This article analyzes cross-country data encompassing 130 countries and regions from 2000 to 2019 to investigate the correlation between financial crises, labor market frictions, and economic volatility. The empirical findings demonstrate that financial crises have a milder impact on real gross domestic product (GDP) in developing countries with flexible labor markets. This trend also applies to non–eurozone developed countries, where labor market flexibility aids crisis mitigation. However, this pattern doesn’t hold for eurozone countries. Further examination of developing nations reveals that those with heightened labor market flexibility tend to experience reduced adverse effects on non-tradable sectors, thereby mitigating the impact on real GDP.

## Introduction

As economic phenomena, financial crises have historically inflicted significant damage on nations and the global economy [1]. They often coincide with substantial bank and business failures, rapid increases in unemployment rates, and related events [2]. This phenomenon prompts extensive research, including modeling and theoretical discussions [3–9]. Given the varied impact on economic activity across countries, numerous studies discuss and summarize factors mitigating the destructive effects of financial crises. These studies note that specific policies adopted by policymakers, such as aggressive monetary easing and proactive fiscal measures, effectively alleviate the negative impact of financial crises [10, 11]. Beyond fiscal and monetary strategies, the labor market also significantly influences a country’s performance during financial crises [12]. Policies and phenomena related to the labor market, such as regulation, unemployment benefits, and wage rigidity, collectively reflect labor market friction and help buffer the shock’s impact [13]. Heightened labor markets friction, like stricter labor regulation and enhanced employment protection, often hinders timely and flexible adjustment, indicating a weaker ability to buffer the shock’s impact [14].

Overall, the studies above primarily delve into theoretical discussions, concluding that increased labor market flexibility can better alleviate the adverse impacts of economic shocks. However, the empirical validation of this relationship needs to be improved. Furthermore, an empirical analysis of how labor market flexibility counteracts adverse shock effects is absent. Therefore, this paper aims to validate the theoretical assertions alongside existing research empirically. It also seeks to uncover the precise channels through which labor market flexibility mitigates the negative consequences of adverse shocks. The article analyzes data from a cross-country panel encompassing 130 countries and regions spanning 2000 to 2019. This analysis explores the empirical connection between financial crises, labor market friction, and economic volatility. The findings highlight that in developing countries, labor market frictions play a moderating role in the correlation between financial crises and economic fluctuations. Precisely, greater labor market flexibility corresponds to a more pronounced ability to mitigate the adverse effects of financial crises on the economy. This correlation remains robust even when introducing different control variables and utilizing diverse indicators as proxies. Moreover, the capacity of labor market flexibility to alleviate the impact of financial crises extends to non–eurozone developed countries, though this conclusion does not apply to eurozone countries. Further investigation into developing countries reveals that those with more adaptable labor markets exhibit a heightened ability to mitigate the impact on non-tradable sectors and, consequently, on real GDP.

This paper presents distinct contributions compared to prior research. Firstly, while previous studies predominantly address the role of labor market flexibility in financial crisis repercussions at a theoretical level, this paper offers empirical validation. The investigation reveals that heightened labor market flexibility corresponds to an enhanced capacity to mitigate the ramifications of financial crises. This empirical validation corroborates conclusions derived from existing theoretical studies. Moreover, the study bridges the gap by applying practical methodologies to test theoretical hypotheses. The paper delves into the impact mechanism, building upon established findings. It demonstrates that heightened labor market flexibility is conducive to attenuating the adverse effects of financial crises on the non-tradable sectors of developing nations. Consequently, this flexibility alleviates the repercussions of financial crises on the real GDP of these economies. This revelation elucidates the impact pathway of labor market flexibility and furnishes policymakers with a more precise roadmap to respond to financial crises. Notably, policymakers should concentrate on deregulating labor in the non-tradable goods sector, offering a strategic means to mitigate the havoc wrought by financial crises effectively.

The rest of the article proceeds as follows. Section 2 provides a review of the literature. Section 3 describes the data and empirical strategy used in the paper. Section 4 presents the main empirical results and mechanism analysis. Section 5 concludes.

## Literature review

Existing research addresses the aftermath of financial crises by discussing the factors that can mitigate their impact, with some studies adopting a policy-oriented perspective. For instance, Mishkin (2009) asserts that a more aggressive approach to easing monetary policy during a financial crisis can reduce interest rates and credit spreads, thus lessening the negative economic consequences. Similarly, expansionary fiscal policy during a crisis is known to counter its adverse effects effectively. Auerbach & Gorodnichenko (2012), drawing on the traditional Keynesian model, highlight that the aggregate supply curve becomes horizontal during a recession, making fiscal expenditures a potent economic stimulus [15]. The examination of government spending multipliers verifies their argument. In a related vein, Kollmann & Roeger (2012) reveal that substantial government backing for the banking sector during crises enhances bank capital and decreases bank spreads, subsequently fostering investment and output growth [16]. Beyond these policy considerations, the role of exchange rate policies is also pivotal. Zeev (2019) establishes that countries adhering to fixed exchange rate regimes experience more significant negative repercussions from adverse global credit supply shocks [17]. Conversely, Zeev (2019) underscores that freely depreciating exchange rates can spur exports via the expenditure-switching mechanism and bolster asset values denominated in foreign currency, easing collateral constraints. Furthermore, labor market policies are gaining prominence in research circles. These policies encompass unemployment benefits, regulations governing hiring and firing practices, and minimum wage regulations, which collectively shape hiring dynamics, production strategies, economic growth trajectories, and societal well-being. Distinct institutional configurations in labor markets across countries give rise to varying degrees of labor market friction, consequently influencing national economic activity. Numerous studies suggest heightened labor market friction correlates with poorer economic performance, including higher unemployment rates [18, 19].

During external shocks, the influence of labor market frictions on the transmission effects of these shocks on the economy becomes even more pronounced. Different degrees of labor market flexibility correspond to varying levels of economic volatility in the face of such shocks. Greater labor market flexibility in a country leads to better mitigation of the impact of adverse shocks, a principle supported by existing research. Two primary reasons underpin this phenomenon. Firstly, when compounded by adverse shocks, labor market frictions heighten uncertainty around employment and decrease workers’ expected income levels, which, in turn, adversely affects economic behavior, impacting consumption, macroeconomic performance, and overall demand. For instance, Boz et al. (2015) emphasize that labor market friction often necessitates a period of unemployment post-dismissal, which during a recession significantly reduces permanent income, elevates employment uncertainty, and prompts increased savings [20]. Donadelli & Grüning (2016) incorporate wage rigidity into an endogenous growth model, revealing that such rigidity restricts households’ ability to react flexibly to productivity shocks, resulting in a heightened incentive for precautionary savings [21]. Ravn & Sterk (2017) assert that labor market frictions lead to lowered worker matching efficiency and diminished job search prospects during unemployment, which, in workers’ eyes, translates to lower anticipated income, fostering more significant savings and impacting overall demand [22]. Secondly, labor market frictions can worsen the adverse impact on firms’ production during unfavorable shocks by raising labor adjustment costs and slowing down labor adjustments, magnifying the negative influence on firms’ output and further accentuating macroeconomic volatility. Caballero et al. (2013) observe that nations with more substantial employment security exhibit slower employment adjustments in response to shocks, ultimately hindering economic growth [23]. Buera et al. (2015) find that credit-constrained enterprises reduce labor demand due to a credit crunch, with unaltered production factors flowing to unconstrained entities through declining factor prices. However, labor market friction disrupts this labor force redistribution, leading to prolonged high unemployment rates. Liao (2021) demonstrates that negative credit impacts coupled with labor market friction restrict labor mobility, leading to an overall decline in employment rates and a reduction in marginal capital productivity [24]. Schmitt-Grohé et al. (2022) contend that capital inflow "sudden stops" dampen a country’s demand, directly lowering non-tradable goods output and labor demand, culminating in elevated unemployment rates in this sector [25].

Consequently, enhancing labor market flexibility effectively mitigates adverse external shock effects. Galí & Monacelli (2016) affirm the role of wage flexibility in alleviating employment-related negative impacts. However, its efficacy may vary among countries with a monetary union versus those with flexible price changes [26]. Georgiadis (2016) highlights that labor market deregulation and similar measures can reduce a nation’s vulnerability to U.S. monetary policy-induced impacts [27].

A literature review reveals numerous studies suggest that countries and regions with greater labor market flexibility experience milder impacts during financial crises. Nevertheless, most of these studies remain theoretical, necessitating empirical validation. Furthermore, there needs to be more exploration into the operational mechanisms underlying labor market flexibility. This article aims to address these issues through empirical analysis.

## Data source and empirical strategy

### Data source

Our dataset includes 130 countries and regions spanning from 2000 to 2019. We source data on various factors, such as government spending as a percentage of GDP, net domestic credit as a portion of GDP, and net inflow of foreign direct investment (FDI) as a fraction of GDP, from the World Bank’s World Development Indicators. Trade openness is gauged based on Miyamoto et al. (2019), using a combination of exports and imports as a share of GDP, with data again derived from the World Development Indicators [28]. Mean years of schooling data are from the United Nations Development Programme’s Human Development Reports Database. In contrast, industry-specific value-added data stem from the United Nations’ National Accounts Main Aggregates Database.

This paper adopts Hardy & Sever (2021) as a reference for the core explanatory variable of financial crises [29], using data on financial crises from Laeven & Valencia (2020) [30]. A country is considered to have experienced a financial crisis if it encounters a banking crisis, sovereign debt crisis, or currency crisis in a given year. Regarding the core explanatory variable of labor market frictions, it’s acknowledged that various indicators like wage flexibility, work hour flexibility, and labor mobility are available. Factors such as wage rigidity, minimum wage levels, and working time regulations affect wage and work hour flexibility, while labor mobility hinges on labor market regulations. Among these factors, wage flexibility and labor mobility have been widely discussed regarding their impact on macroeconomic volatility. Given that previous studies have left some gaps in the empirical measurement of wage flexibility, and considering our definition of labor market frictions in conjunction with Di Tella & MacCulloch (2005) [18] and Du & Liu (2015), we concentrate on analyzing the effect of labor market regulation in this study [31]. Specifically, we use the Hiring and Firing Regulation indicator (category 5Bii) from the Fraser Institute’s Economic Freedom of the World Database to gauge labor market frictions. This indicator reflects the level of labor regulation within each country or region, assigning higher scores to places with less regulated hiring and firing practices and a more flexible labor market. Furthermore, in the subsequent robustness tests, we employ "Hiring and Firing Practices" (an indicator from the Global Competitiveness Report provided by the World Economic Forum) as an additional proxy indicator for labor market flexibility. This indicator mirrors the degree of regulation in a country’s hiring and firing practices, with higher values signifying less regulation and greater labor market flexibility.

Regarding the dependent variable, this paper adopts Romer & Romer’s (2019) approach and selects real GDP as the variable of interest. Real GDP represents the market value of all final products in the current year, calculated using the previous year’s prices as the base period. This choice allows for a more comprehensive examination of the influence of product output fluctuations. Data for real GDP are from the International Monetary Fund’s World Economic Outlook Database. Table 1 displays descriptive statistics for the variables.

**Table 1:** Descriptive statistics.

Variable	Observations	Mean	Standard deviation	Minimum value	Maximum value	Data source
Labor market flexibility	2,276	4.634	1.322	0	8.8	EFW^a^
Financial crisis (0–1)	2,276	0.070	0.256	0	1	Laeven and Valencia (2020)
ln (Real GDP^b^)	2,276	13.423	2.723	6.755	33.830	WEO^c^
Government consumption / GDP	2,155	0.160	0.054	0.010	0.508	WDI^d^
Net domestic credit / GDP	1,965	0.511	0.382	-0.548	1.496	WDI
Level of openness to trade	2,086	0.768	0.319	0.196	1.646	WDI
Mean years of schooling	2,273	8.610	3.029	1.308	14.152	HDR^e^
FDI^f^ net inflows / GDP	2,244	0.043	0.059	-0.283	0.474	WDI
Labor market flexibility (in robustness tests)	1,657	3.827	0.751	1.802	6.106	GCR^g^
Value added(non-tradable sector) / GDP	2,290	0.614	0.097	0.189	0.871	NAMAD^h^
Value added in tradable sector / GDP	2,290	0.309	0.113	0.056	0.671	NAMAD
Value added (ISIC^i^ F) / GDP	2,290	0.056	0.025	0.006	0.214	NAMAD
Value added (ISIC G-H)/ GDP	2,290	0.141	0.039	0.028	0.305	NAMAD
Value added (ISIC I) / GDP	2,290	0.080	0.025	0.019	0.180	NAMAD
Value added (ISIC J-P) / GDP	2,290	0.337	0.092	0.071	0.619	NAMAD

^a^ EFW = World Economic Freedom Database.

^b^ GDP = gross domestic product.

^c^ WEO = World Economic Outlook Database.

^d^ WDI = World Development Indicators.

^e^ HDR = Human Development Reports Database.

^f^ FDI = foreign direct investment.

^g^ GCR = Global Competitiveness Report.

^h^ NAMAD = National Accounts Main Aggregates Database.

^i^ ISIC = International Standard Industrial Classification.

### Empirical strategy

In this subsection, we develop the empirical model for our subsequent analysis. We employ the local projection method introduced by Jordà (2005), which uses ordinary least squares to estimate the effects of the explanatory variable on the dependent variable across various future periods through multi-step regression [32]. Unlike linear panel regression models, this approach enables the exploration of intertemporal relationships between variables. Compared with vector autoregressive models, which also estimate impulse response functions, the local projection method avoids the need for subjective parameter constraints in the coefficient matrix estimation. This advantage circumvents the limitations associated with vector autoregressive models. Hence, we employ the local projection method to investigate whether labor market flexibility can alleviate the economic impact of a financial crisis both during and after the crisis.


$$\begin{array}{ll} y_{i,t+h}-y_{i,t-1} & =\alpha_{i}^{h}+\beta^{h}crisis_{i,t}+\gamma^{h}flexibility_{i,t}+\delta^{h}\left(crisis_{i,t}\times flexibility_{i,t}\right)\\ & +\mu^{h}\Delta y_{i,t-1}+\theta^{h}crisis_{i,t-1}+\tau^{h}flexibility_{i,t-1}\\ & +\varphi^{h}(crisis_{i,t-1}\times flexibility_{i,t-1})+X_{i,t}+\varepsilon_{i,t}, \end{array}$$ (1)

Our specification is as follows:

The equation involves the dependent variable, *y*_*i*,*t*+*h*_, which signifies the real GDP of country i in year t+h. Here, h ranges from 0 to 4, capturing the real GDP impact from time t onwards over the subsequent four years. The difference *y*_*i*,*t*+*h*_−*y*_*i*,*t*-1_ quantifies the real GDP change in country i during year t+h compared to year t-1. On the equation’s right side, the explanatory variable crisis_i,t_, functions as a binary indicator, denoting the occurrence of a financial crisis in country i during year t. A crisis triggers the variable to be 1; otherwise, 0. The coefficient *β*^*h*^ before the variable elucidates the crisis’s influence in period t on real GDP in period t+h. Due to the unpredictable nature of financial crises, they are treated as exogenous shocks to investigate labor market friction’s impact on economic volatility. This approach sidesteps the endogeneity issue stemming from the reverse causality between labor market friction and general economic downturns. Meanwhile, flexibility_,t_ characterizes the labor market flexibility in country i during year t, with higher values signifying greater flexibility.

To examine labor market flexibility’s role in the repercussions of financial crises, we introduce the interaction term *crisis*_,*t*_ × *flexibility*_*i*,*t*_ between the financial crisis and labor market flexibility variables. This interaction term illuminates labor market flexibility’s moderating effect on the correlation between financial crises and real GDP. Moreover, for a comprehensive comprehension of year t’s labor market flexibility’s influence on the current financial crisis’s impact on immediate and future economic conditions, the equation integrates controls for crisis inception and economic circumstances before year t. It incorporates lag terms representing financial crises, real GDP growth, labor market flexibility, and the interaction between labor market flexibility and financial crises as explanatory factors. Given the 20-year sample period, we include one lag of the abovementioned variables to ensure an ample number of observations. The country fixed effect, $\alpha_{i}^{h}$, accounts for unobservable factors persisting overtime at the country level. An array of controls, *X*_*it*_, encompasses pivotal GDP-impacting variables like general government final consumption expenditure (% of GDP), mean years of schooling, net domestic credit (% of GDP), trade openness level, and FDI net inflows (% of GDP). We incorporate these controls in the robustness checks to validate the empirical results’ robustness. Lastly, Finally, *ε*_*i*,*t*_ is the error term.

## Empirical analysis

### Basic empirical results

**Fig 1:**
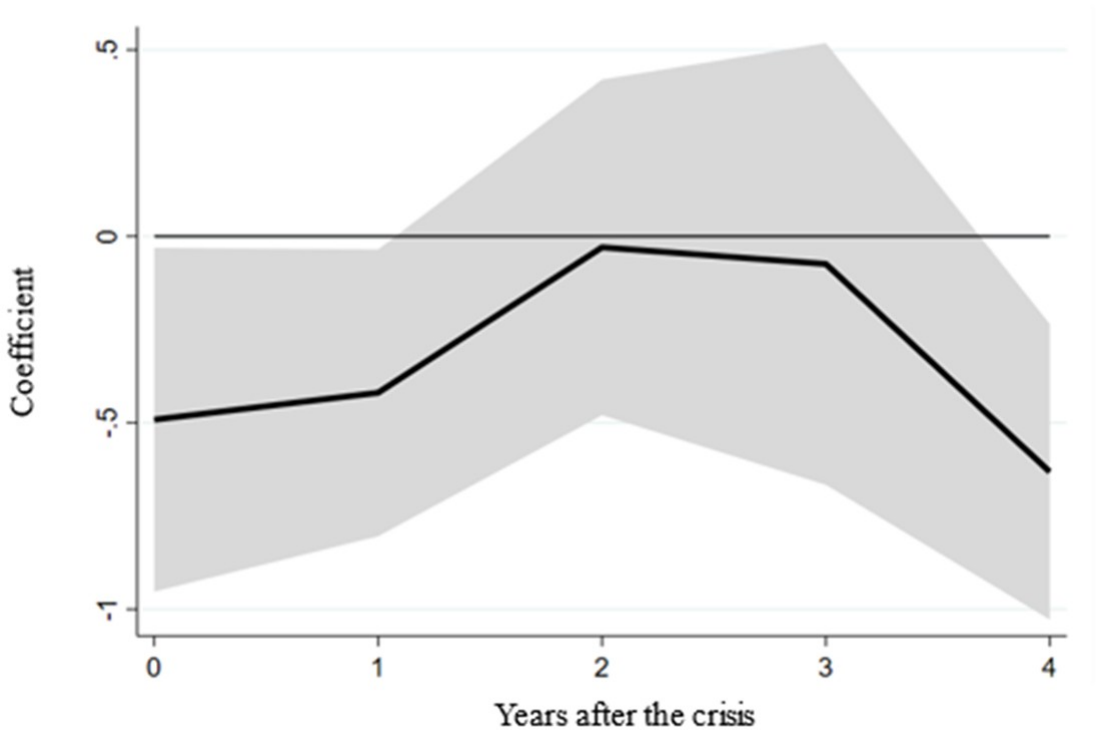
The impact of the financial crisis on the real GDP of the full sample. Note: The figure illustrates how the financial crisis affected real GDP at various points within the full sample period. The black line signifies the estimated coefficients linked to the financial crisis. At the same time, the shaded region depicts the confidence interval, accounting for a range of plus or minus 1.645 standard deviations of the coefficient.

In line with the outlined empirical framework, our initial step involves examining the relationship between financial crises and real GDP before delving into the potential influence of a country’s labor market friction on crisis impact. This preliminary assessment enables us to observe the empirical implications of financial crises. Fig 1 presents the coefficients of financial crises, denoted as β^h^, across different time horizons after estimating the above equation using the full sample. The horizontal axis corresponds to the years following a financial crisis. In contrast, the vertical axis illustrates the coefficients of financial crises, β^h^, quantifying their effect on real GDP at horizon h post-crisis. The figure demonstrates that the coefficient of financial crises, β^h^, exhibits significant negativity up to horizon 2, indicating an immediate adverse impact of financial crises on the global economy.

**Fig 2:**
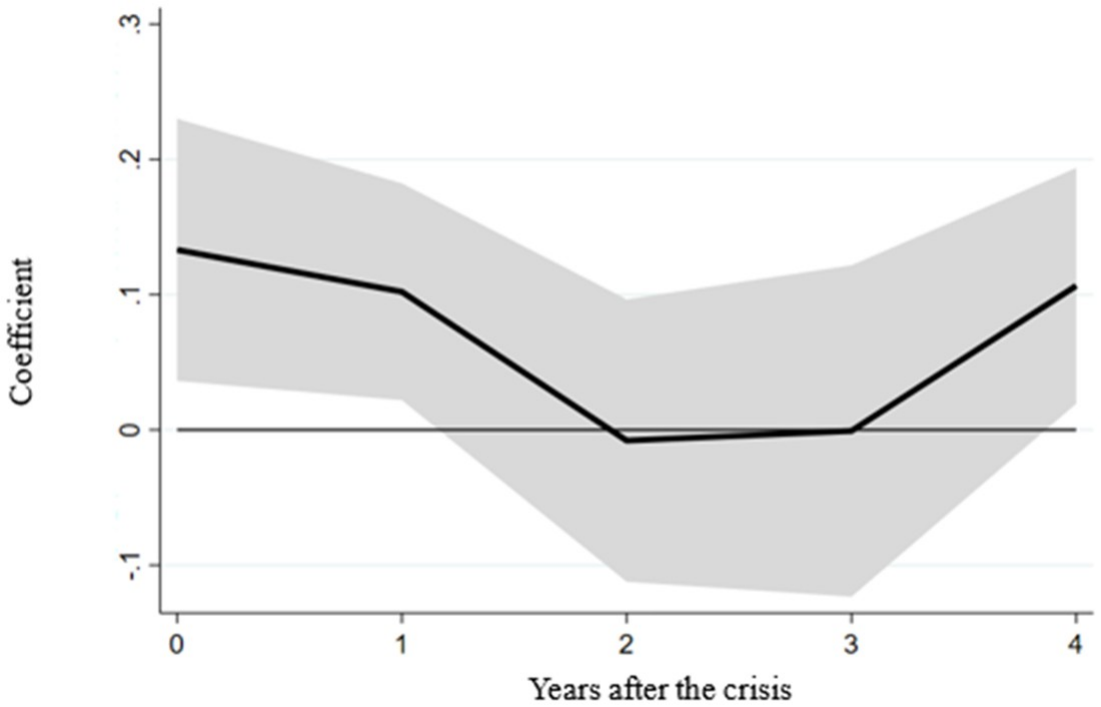
The effect of labor market flexibility on the real GDP of the entire sample. Note: The figure depicts the influence of the interaction between the financial crisis and labor market flexibility on real GDP during various periods within the full sample. The black line denotes the estimated coefficients for this interaction. At the same time, the shaded region indicates the confidence interval, accounting for a range of plus or minus 1.645 standard deviations of the coefficient.

Building upon the outlined framework, we investigate the potential mitigating role of labor market flexibility on the impact of financial crises within the sample. The results of this analysis are depicted in Fig 2, showcasing the estimated coefficients (denoted as δ^h^) of the interaction term specified in the econometric model across different horizons. As illustrated in the figure, the coefficient exhibits significant positivity in the year immediately following a financial crisis. This observation implies that countries with more excellent labor market flexibility experience relatively milder declines in real GDP during financial crises.

**Fig 3:**
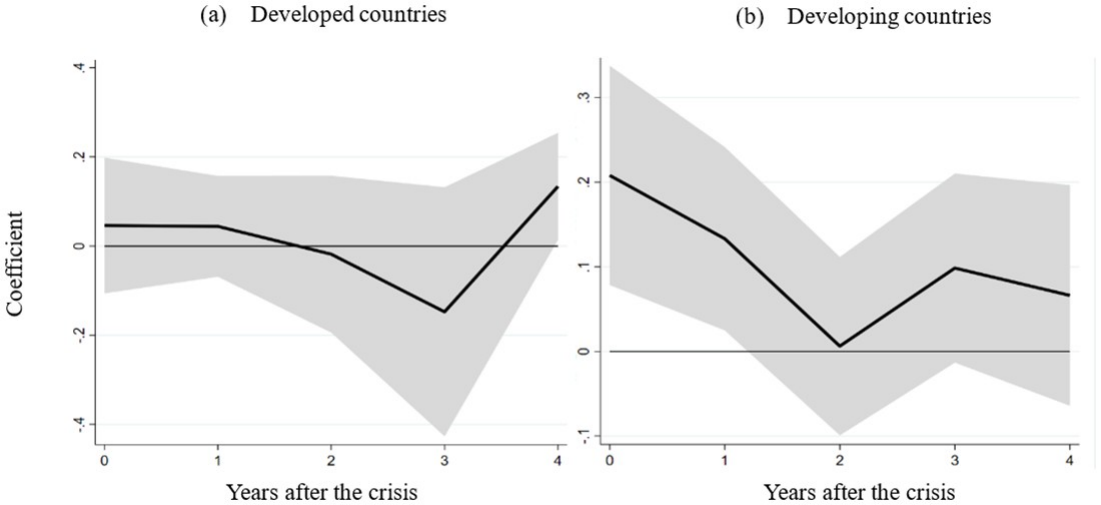
The effects of the interaction between financial crisis and labor market flexibility on real GDP in developed and developing countries.

Taking into account the developmental disparities between developed and developing nations, we partition the dataset to explore whether labor market flexibility can mitigate the effects of financial crises in countries of varying development levels. In Panel (a) of Fig 3, the temporal evolution of the interaction coefficient δ^h^ between labor market flexibility and financial crises is depicted. This analysis uses the regression model outlined in section 3 and focuses on the sample of developed countries. The figure illustrates that the interaction term coefficient fails to reach statistical significance, implying that labor market flexibility in developed countries does not appear to alleviate the impact of financial crises. Shifting to Panel (b) of Fig 3, the coefficient of the interaction term is presented based on the regression analysis performed on the dataset of developing countries. Notably, the figure demonstrates that the coefficient of the interaction term exhibits significant positivity within the first year following a crisis. These results suggest that developing nations with more flexible labor markets experience comparatively milder repercussions during financial crises. Given that the moderating influence of labor market flexibility is exclusively observable in the sample of developing countries, the forthcoming robustness assessments and mechanism analyses will be centered on this subset of nations.

**Fig 4:**
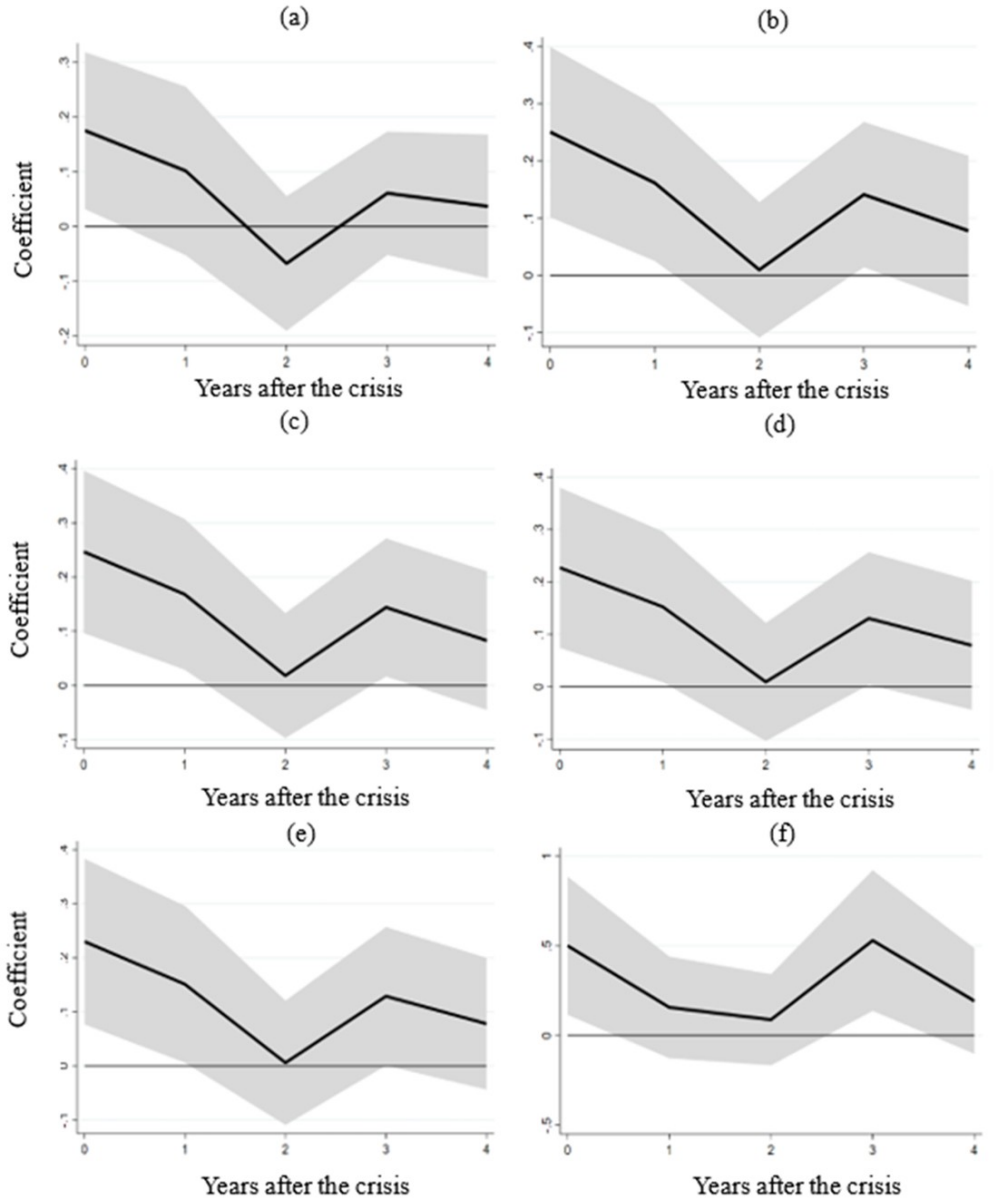
Robustness checks. Note: The figure illustrates the impact of the interaction term between the financial crisis and labor market flexibility on real GDP in developing countries. (a) excludes the data for 2008 and 2009, while (b) incorporates government consumption as a control variable in the regression. In (c), government consumption and mean years of schooling are included as control variables. Moving to (d), the regression accounts for government consumption, mean years of schooling, and net domestic credit as control variables. (e) extends this analysis by adding government consumption, mean years of schooling, net domestic credit, trade openness level, and net inflows of foreign direct investment as control variables. Finally, (f) presents the results when substituting the labor market flexibility indicator.

In light of the 2008 subprime mortgage crisis and its subsequent global impact, we exclude data for 2008 and 2009 from the analysis of developing countries. This step allows us to scrutinize whether extreme values influence labor market flexibility’s effects. Notably, the impulse response in panel (a) of Fig 4 confirms that the interaction term’s coefficient remains significantly positive within a year following a crisis. Numerous factors intricately intertwine with a nation’s economic landscape. Fiscal policy, for instance, closely corresponds with economic conditions, wherein fiscal expansion during recessions can effectively stimulate the economy. A country’s education level plays a significant role in economic dynamics, as a well-educated population and heightened labor productivity catalyze economic progress [33, 34]. Additionally, the research delves into GDP growth determinants encompassing credit, trade, and investment aspects. Credit inflows tend to invigorate economic activities [35], trade openness fosters economic growth through technological diffusion and resource accessibility [36, 37], while foreign direct investment drives economic expansion by directly accumulating factors [38]. Hence, control variables, including government consumption (as a share of GDP), education level, net domestic credit (as a share of GDP), trade openness, and net FDI inflow (as a share of GDP), are progressively introduced to the original equation. Fig 4 demonstrates that adding these control variables maintains the significantly positive coefficients of the interaction term. Furthermore, aligning with the abovementioned labor market flexibility definition, we substitute the initial variable with the "hiring and firing practices" index from the Global Competitiveness Report. This index accurately gauges the extent of regulations surrounding worker hiring and firing. Panel (f) in Fig 4 portrays the outcomes post this substitution, reaffirming the steadfastness of the positive relationship between the interaction term and real GDP. The results underscore the resilience of the positive connection between the interaction term of labor market flexibility and financial crises and real GDP within developing countries.

Schmitt-Grohé et al. (2022) suggest that a combination of downward nominal wage rigidity and fixed exchange rates can lead to involuntary unemployment in the non-tradable sector when capital inflows suddenly cease. This inference implies that all else being equal, enhancing labor market flexibility in nations with floating exchange rate systems results in more adaptable real wages and a more pronounced impact than in countries with fixed exchange rate systems. This finding aligns with Galí & Monacelli’s (2016) conclusions. Based on the preceding empirical outcomes, labor market flexibility does not appear to mitigate the consequences of financial crises in developed countries. However, it’s noteworthy that several developed nations are part of the eurozone, utilizing the euro as their unified currency and maintaining fixed exchange rates within the member states. Given these insights and the euro zone’s dynamics, a pertinent question emerges: Could the nonsignificant findings in developed countries be attributed to the role of labor market flexibility in eurozone nations? To address this, we can partition the sample of developed countries into those within the eurozone and those outside it. By analyzing labor market flexibility’s impact on these two groups separately, we aim to ascertain any contrasting empirical results.

**Fig 5:**
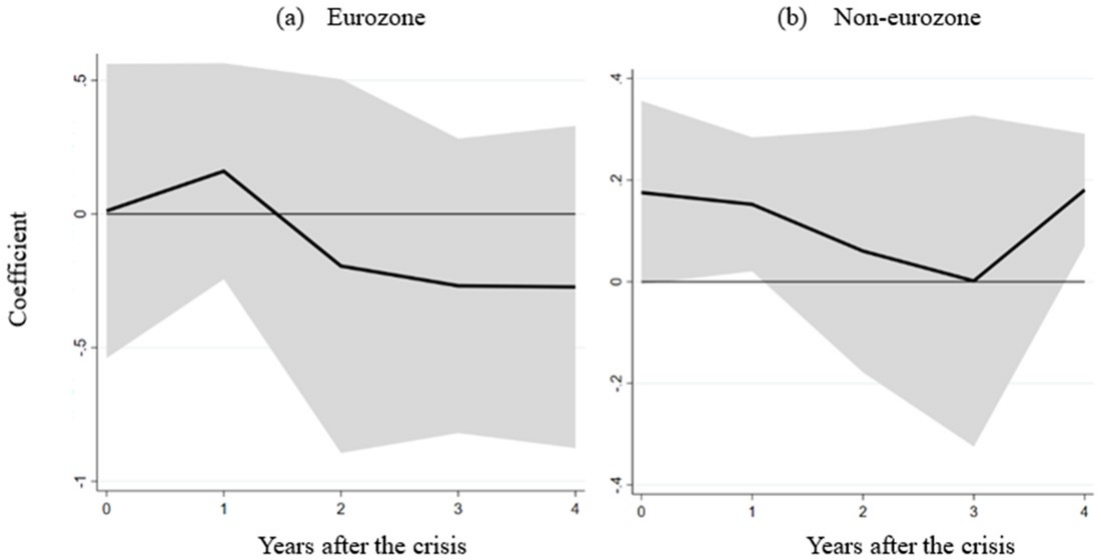
The role of labor market flexibility in the eurozone and non–eurozone countries. Note: (a) and (b) depict the effect of the interaction term between the financial crisis and labor market flexibility on real GDP in the eurozone and non–eurozone countries.

Our empirical examination unfolds in Fig 5, where the estimated interaction term values for the eurozone and non-eurozone countries are showcased across various horizons (h), utilizing the regression model elucidated in section 3. Panel (a) portrays the interaction term’s effect on real GDP for eurozone countries—noticeably, no significant impact is discerned. Conversely, panel (b) unveils that non-eurozone countries exhibit a substantially positive influence from the interaction term within the initial year post-crisis. This pattern signifies that financial crises exact a milder toll on real GDP in non-eurozone countries with more flexible labor markets. Notably, most non-eurozone countries operate under floating or flexible exchange rate systems. The empirical findings underscore the heterogeneous role of labor market flexibility in mitigating crisis effects across different exchange rate regimes. In non-eurozone countries, labor market flexibility is pivotal in cushioning the impact of the crisis. In stark contrast, within the eurozone—marked by a common currency—such a pattern does not manifest. This analysis substantiates earlier conclusions that labor market flexibility significantly eases crisis-related damage in countries with floating exchange rates.

### Mechanism analysis

Theoretical studies underscore the connection between labor market deregulation and the adaptable adjustment of the workforce during external shocks, facilitating employment and output. Some investigations start by exploring wage rigidity’s effects through theoretical models. Instead of free wage adjustments, downward wage rigidity can hinder labor wages from adapting efficiently to a crisis-induced drop in labor demand. This mismatch can lead to an oversupply of labor, resulting in unemployment at the existing wage level [39]. Further theoretical research highlights how factors like employment protection and labor matching friction can intensify unemployment after adverse shocks. In congruence with these theoretical findings, our prior empirical analysis unveils that labor market flexibility significantly mitigates the adverse impact of financial crises on developing countries. This alignment between theory and empirical outcomes strengthens our conclusions. Now, we focus on identifying sectors within developing economies where labor market flexibility yields such an impact. Could labor market deregulation drive greater flexibility, stimulating employment and output adjustments under crisis, particularly in tradable and non-tradable sectors? To address these inquiries, we adapt our previous regression by replacing the dependent variable with tradable or non-tradable sector value added (% of GDP). Correspondingly, we adjust the explanatory variable’s first-order lag term to the change in value added of the tradable or non-tradable sector (% of GDP). By doing so while keeping other variables constant and estimating the equation, we elucidate the influence of the interaction term between financial crises and labor market flexibility on these sectors. This replacement empowers us to empirically explore the specific mechanism of labor market flexibility’s impact.

**Fig 6:**
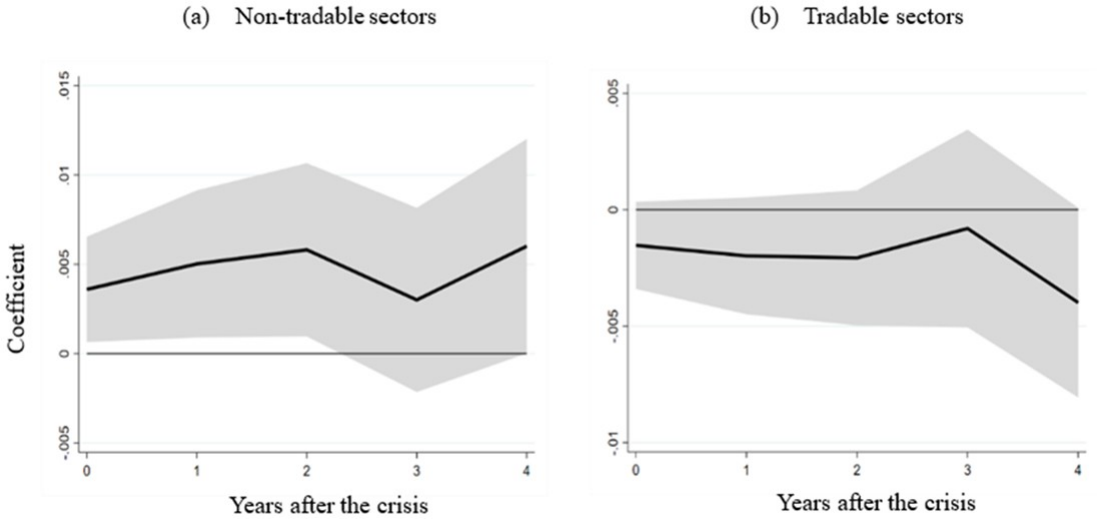
The impact of labor market flexibility on the non-tradable and tradable sectors of developing countries. Note: (a) and (b) demonstrate the influence of the interaction term between the financial crisis and labor market flexibility on the value added (% of gross domestic product) within developing countries’ non-tradable and tradable sectors.

In panel (a) of Fig 6, the interaction term coefficient between financial crises and labor market flexibility within the non-tradable sector is significantly positive within two years post-crisis. This result implies that countries with more adaptable labor markets experience a milder negative effect on non-tradable sectors. In contrast, the interaction term coefficient displayed in panel (b) is insignificant, indicating that labor market flexibility doesn’t notably alleviate the financial crisis’s impact on the tradable sector. Our empirical analysis thus discerns labor market flexibility’s efficacy in mitigating non-tradable sector repercussions. However, the extent of this mitigation across specific industries within the non-tradable sector remains to be explored.

To address these queries, we modify the equation from section 3 by substituting the dependent variable with the value added of specific industries within the non-tradable sector (% of GDP). In tandem, we replace the first-order lag term of the change in real GDP within the explanatory variable with the first-order lag term of the difference in the value-added of the chosen industry (% of GDP). Data regarding industry value added are from the United Nations’ National Accounts Main Aggregates Database. This database classifies industries under the International Standard Industrial Classification (ISIC) code across six categories. Within the non-tradable sector, the database encompasses the value added-to-GDP ratio for four categories: ISIC F (construction), ISIC G-H (wholesale and retail, hotels and restaurants), ISIC I (transportation, storage, and communications), and ISIC J-P (other industries, encompassing financial intermediation, real estate, renting and business activities, public administration and defense, compulsory social security, education, health and social work, other community services, and social and personal service activities). We estimate the resulting equation by sequentially substituting the real GDP variable with the value added of these four categories.

**Fig 7:**
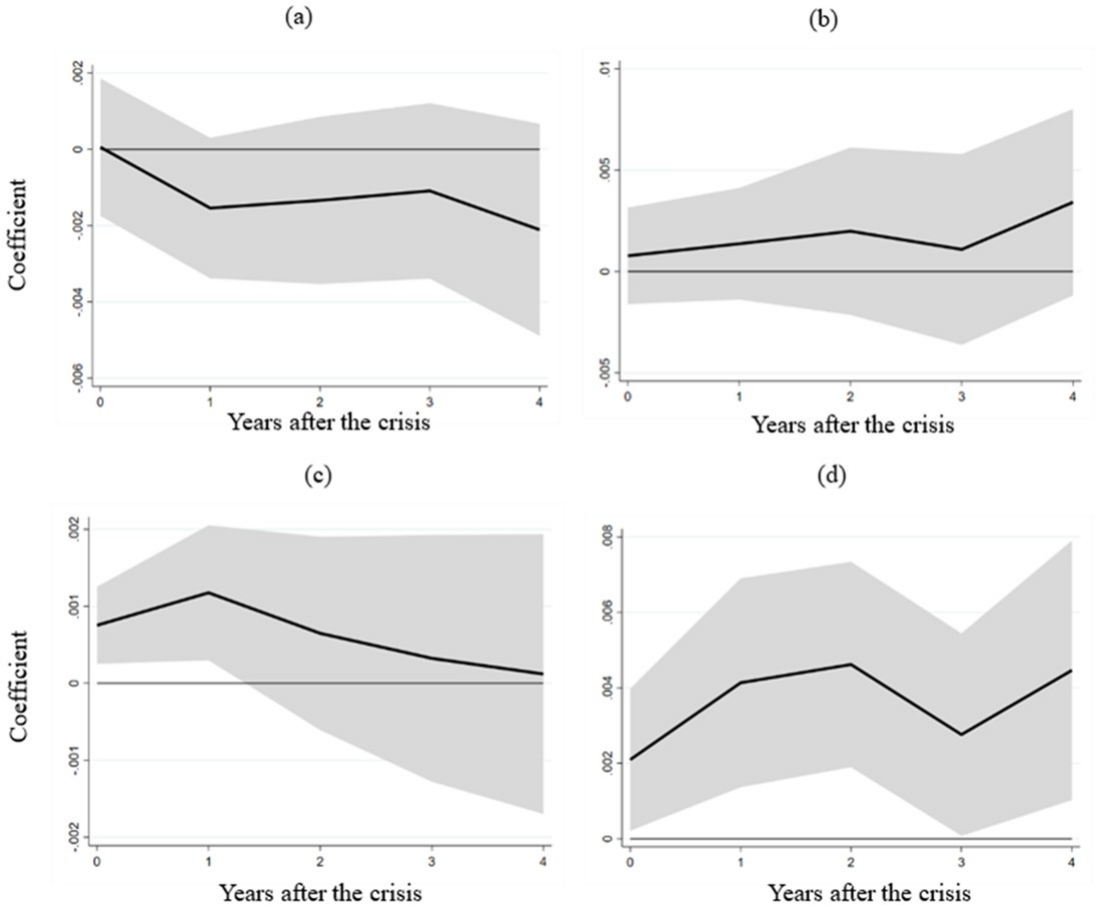
The impact of labor market flexibility on selected industries in developing countries. Note: The figure illustrates the influence of the interaction term between the financial crisis and labor market flexibility on the value added (% of gross domestic product) within distinct industries in developing countries. (a) displays the outcomes for the construction industry (ISIC F). Moving to (b), we observe the findings for the wholesale, retail trade, restaurants, and hotels industries (ISIC G-H). Shifting focus to (c), we can see the results for the transport, storage, and communication industries (ISIC I). Finally, (d) reveals the outcomes for the remaining industries (ISIC J-P).

Fig 7 presents the outcomes of these interactions between labor market flexibility and financial crises following the aforementioned variable substitution and estimation. Panels (a) and (b) exhibit nonsignificant interaction terms, suggesting that labor market flexibility fails to alleviate the impact of financial crises on value-added within the construction (ISIC F) and wholesale, retail, hotels, and accommodations industries (ISIC G-H). Contrarily, panels (c) and (d) unveil positive and significant interaction term effects on value added in the transportation, storage, and communication industries (ISIC I) and other non-tradable sector industries (ISIC J-P). These results indicate that more flexible labor markets in developing countries lead to diminished adverse impacts on these industries during financial crises. Consequently, these findings illuminate the specific pathway through which labor market flexibility mitigates financial crisis repercussions.

## Conclusion

Limited empirical research exists on whether labor market flexibility can mitigate the impact of financial crises on the economy. Empirical research concerning the potential of labor market flexibility to alleviate financial crisis impacts on economies has been limited. This study utilizes cross-country panel data to ascertain that enhanced labor market flexibility in developing nations can lessen the detrimental effects of financial crises, with substantial robustness in conclusion. The analysis underscores that labor market flexibility doesn’t counteract the harms inflicted by financial crises among developed countries in the eurozone. At the same time, this mechanism does function for countries outside the euro. Furthermore, exploring crisis effects on the non-tradable sector reveals that heightened flexibility diminishes severity, a pattern that doesn’t extend to the tradable sector.

By forging connections between labor market frictions, financial crises, and economic volatility, this study establishes that labor market deregulation aids in mitigating the adverse consequences of financial crises. This paper offers practical guidance in the following areas. Firstly, policymakers should carefully consider the role of labor market flexibility in countering the impact of financial crises. They should adjust labor market regulations appropriately, enhance adaptability to crises, and improve employment and output levels. Secondly, the analysis within this paper demonstrates that greater labor market flexibility is associated with a heightened ability to mitigate shocks to the non-traded goods sector and, subsequently, shocks to real GDP. Policymakers should prioritize assessing the influence of financial crises on the non-traded goods sector, which involves ensuring the labor force can achieve unhindered inter-industry mobility during crises. This action aims to minimize the detrimental effects of financial crises on the non-traded goods sector, thereby alleviating the adverse economic shocks.

## Supporting information

S1 Data (DTA)

S1 File The developed countries, tradable and non-tradable sectors included in this paper. (PDF)

S2 File (DO)
