# Supplementary material for: Financial crisis, labor market frictions, and economic volatility
Source: PLoS One. 2023 Sep 28;18(9):e0291106. doi: 10.1371/journal.pone.0291106 (PMC10538789; doi:10.1371/journal.pone.0291106)
Supplement: S1 File — (PDF) [file pone.0291106.s002.pdf]

S1 File. The developed countries, tradable and non-tradable sectors included in this paper

The developed countries included in this paper encompass Australia, Austria, Belgium, Canada, Cyprus, Denmark, Finland, France, Germany, Greece, Iceland, Ireland, Italy, Israel, Japan, Luxembourg, Malta, the Netherlands, New Zealand, Norway, Portugal, Singapore, Slovenia, Spain, Sweden, Switzerland, the United Kingdom, and the United States.

The tradable sectors included in this paper encompass activities like agriculture, hunting, forestry, fishing, mining, quarrying, manufacturing, and the provision of electricity, gas, and water. On the other hand, the non-tradable sectors comprise construction, wholesale and retail trade, hotels and restaurants, transportation, storage, and communication services, financial intermediation, real estate, renting and business activities, public administration, defense, compulsory social security, education, health, social work, other community services, and social and personal service activities.
